# Supplementary material for: Screening accuracy and cut-offs of the Polish version of Communication and Symbolic Behavior Scales-Developmental Profile Infant-Toddler Checklist
Source: PLoS One. 2024 Aug 9;19(8):e0299618. doi: 10.1371/journal.pone.0299618 (PMC11315298; doi:10.1371/journal.pone.0299618)
Supplement: S1 File — (PDF) [file pone.0299618.s001.pdf]

CSBS DP™

Kwestionariusz CSBS-DP ITC

Communication and Symbolic Behavior Scales – Developmental Profile Infant/Toddler Checklist

Imię i nazwisko dziecka: **Data urodzenia dziecka:**

W którym tygodniu ciąży miał miejsce poród: **Data wypełnienia kwestionariusza:**

Osoba wypełniająca: **Pokrewieństwo z dzieckiem:**

Instrukcja dla opiekunów: Kwestionariusz ma na celu identyfikację różnych aspektów rozwoju dzieci i niemowląt. Wiele zachowań pojawiających się przed wykształceniem umiejętności mowy może wskazywać na ewentualne trudności w nauce mowy lub innych trudności rozwojowych u dziecka. Poniższy kwestionariusz rodzic/opiekun dziecka powinien wypełnić gdy dziecko jest **pomiędzy 6 a 24 miesiącem życia** w celu ustalenia czy konieczne są dalsze konsultacje dotyczące rozwoju dziecka. Opiekun może być rodzicem lub inną osobą, która codziennie opiekuje się dzieckiem. Należy zaznaczyć odpowiedzi, które najtrafniej opisują zachowanie dziecka. W razie wątpliwości proszę zaznaczyć najtrafniejszą opinię bazując na własnym doświadczeniu. **U dzieci w danym wieku niekoniecznie muszą pojawiać się wszystkie wymienione zachowania.**

**Emocje i kontakt wzrokowy**					
1. Czy wiesz, kiedy Twoje dziecko jest szczęśliwe, a kiedy jest zdenerwowane?	◯ Jeszcze nie	◯ Czasami	◯ Często		
2. Kiedy Twoje dziecko bawi się zabawkami, czy spogląda ma ciebie, aby zobaczyć, czy je obserwujesz?	◯ Jeszcze nie	◯ Czasami	◯ Często		
3. Czy Twoje dziecko uśmiecha lub śmieje się patrząc na ciebie?	◯ Jeszcze nie	◯ Czasami	◯ Często		
4. Kiedy patrzysz i wskazujesz na zabawkę po drugiej stronie pokoju, czy Twoje dziecko patrzy na nią?	◯ Jeszcze nie	◯ Czasami	◯ Często		
**Komunikacja**					
5. Czy Twoje dziecko informuje cię, że potrzebuje pomocy lub chce mieć przedmiot poza jego zasięgiem?	◯ Jeszcze nie	◯ Czasami	◯ Często		
6. Kiedy nie zwracasz uwagi na swoje dziecko, czy stara się ono zwrócić twoją uwagę?	◯ Jeszcze nie	◯ Czasami	◯ Często		
7. Czy Twoje dziecko stara się cię rozśmieszyć?	◯ Jeszcze nie	◯ Czasami	◯ Często		
8. Czy Twoje dziecko próbuje skłonić cię do zauważenia interesujących rzeczy - tylko po to, byś spojrzał(a) na daną rzecz, a nie po to, abyś coś z nią zrobiła?	◯ Jeszcze nie	◯ Czasami	◯ Często		
**Gesty**					
9. Czy Twoje dziecko podnosi przedmioty i daje je tobie?	◯ Jeszcze nie	◯ Czasami	◯ Często		
10. Czy Twoje dziecko pokazuje ci przedmioty, nie dając ci ich?	◯ Jeszcze nie	◯ Czasami	◯ Często		
11. Czy Twoje dziecko macha, by powitać inne osoby?	◯ Jeszcze nie	◯ Czasami	◯ Często		
12. Czy Twoje dziecko wskazuje palcem na różne przedmioty?	◯ Jeszcze nie	◯ Czasami	◯ Często		
13. Czy Twoje dziecko kiwa głową, aby zasygnalizować „*tak*”?	◯ Jeszcze nie	◯ Czasami	◯ Często		
**Dźwięki**					
14. Czy Twoje dziecko używa dźwięków lub słów, aby zwrócić na siebie uwagę lub poprosić o pomoc?	◯ Jeszcze nie	◯ Czasami	◯ Często		
15. Czy Twoje dziecko łączy pojedyncze dźwięki tworząc takie zbitki jak: *mama, gaga, papa, dada*?	◯ Jeszcze nie	◯ Czasami	◯ Często		
16. Ile z poniższych dźwięków wymawia Twoje dziecko: *ma, na, ba, da, ga, ka, la, ja, ta, pa?*	◯ Żadnego	◯ 1-2	◯ 3-4	◯ 5-8	◯ ponad 8
**Słowa**					
17. Jak wiele słów Twoje dziecko wymawia w sposób, który rozpoznajesz? (np. jak *am am* na jedzenie, *tote* na kotek itp.)	◯ 0	◯ 1-3	◯ 4-10	◯ 11-30	◯ powyżej 30
18. Czy Twoje dziecko składa dwa słowa razem (na przykład: *daj piciu, tata pa pa*)?	◯ Jeszcze nie	◯ Czasami	◯ Często		
**Rozumienie**					
19. Kiedy wołasz swoje dziecko po imieniu, czy reaguje patrząc na Ciebie, lub obracając się w Twoją stronę?	◯ Jeszcze nie	◯ Czasami	◯ Często		
20. Ile różnych słów lub zwrotów dziecko rozumie bez gestów? Na przykład, jeśli powiesz "*gdzie jest brzuszek*", „*gdzie jest mama*", „*daj piłkę*" lub „*chodź do taty*" (bez wskazywania), czy dziecko odpowiednio reaguje?	◯ Na żadne	◯ 1-3	◯ 4-10	◯ 11-30	◯ powyżej 30
**Użycie przedmiotów**					
21. Czy Twoje dziecko wykazuje zainteresowanie zabawą różnymi przedmiotami?	◯ Jeszcze nie	◯ Czasami	◯ Często		
22. Ile z następujących przedmiotów Twoje dziecko używa w odpowiedni sposób: kubek, butelka, miska, łyżka, grzebień/szczotka, szczoteczka do zębów, ręcznik, piłka, zabawki: samochód, telefon.	◯ Żadnego	◯ 1-2	◯ 3-4	◯ 5-8	◯ ponad 8
23. Ile klocków (lub kółek) Twoje dziecko potrafi ułożyć jeden na drugim?	◯ Żadnego	◯ 2 klocki	◯ 3-4 klocki	◯ 5 lub więcej	
24. Czy Twoje dziecko odgrywa scenariusze w zabawie (np. karmi pluszaka, kładzie lalkę spać, wkłada figurkę do samochodu)	◯ Jeszcze nie	◯ Czasami	◯ Często		

Czy masz jakieś obawy dotyczące rozwoju dziecka? ◯ tak ◯ nie **Jeśli tak, proszę opisz je na odwrocie.**

Wersja oryginalna: Amy M. Wetherby, Barry M. Prinzant © 2002, Paul H. Brookes Publishing Co, Inc.
Wersja polska za zgodą autorów wersji oryginalnej: Mateusz Sobieski, www.spojrzecwoczy.pl
