# Supplementary material for: Screening accuracy and cut-offs of the Polish version of Communication and Symbolic Behavior Scales-Developmental Profile Infant-Toddler Checklist
Source: PLoS One. 2024 Aug 9;19(8):e0299618. doi: 10.1371/journal.pone.0299618 (PMC11315298; doi:10.1371/journal.pone.0299618)
Supplement: S2 File — Re-translated (from Polish to English) Polish version of CSBS-DP ITC. (PDF) [file pone.0299618.s002.pdf]

CSBS DP

CSBS-DP ITC Questionnaire

Communication and Symbolic Behavior Scales – Developmental Profile Infant/Toddler Checklist

Child's name and surname: | **Date of child's birth:**

In what week of pregnancy did the birth take place: | **Date filled out:**

Filled out by: | **Relationship to child:**

Instructions for caregivers: This Checklist is designed to identify different aspects of development in infants and toddlers. Many behaviors that develop before children talk may indicate whether or not a child will have difficulty learning to talk. This Checklist should be completed by a caregiver when the child is between 6 and 24 months of age to determine whether a referral for an evaluation is needed. The caregiver may be either a parent or another person who nurtures the child daily. Please check all the choices that best describe your child's behavior. If you are not sure, please choose the closest response based on your experience. **Children at your child's age are not necessarily expected to use all the behaviors listed.**

**Emotion and Eye Gaze**					
1. Do you know when your child is happy and when your child is upset?		◯ Not Yet	◯ Sometimes		◯ Often
2. When your child plays with toys, does he/she look at you to see if you are watching?		◯ Not Yet	◯ Sometimes		◯ Often
3. Does your child smile or laugh while looking at you?		◯ Not Yet	◯ Sometimes		◯ Often
4. When you look at and point to a toy across the room, does your child look at it?		◯ Not Yet	◯ Sometimes		◯ Often
**Communication**					
5. Does your child let you know that he/she needs help or wants an object out of reach?		◯ Not Yet	◯ Sometimes		◯ Often
6. When you are not paying attention to your child, does he/she try to get your attention?		◯ Not Yet	◯ Sometimes		◯ Often
7. Does your child do things just to get you to laugh?		◯ Not Yet	◯ Sometimes		◯ Often
8. Does your child try to get you to notice interesting objects—just to get you to look at the objects, not to get you to do anything with them?		◯ Not Yet	◯ Sometimes		◯ Often
**Gestures**					
9. Does your child pick up objects and give them to you?		◯ Not Yet	◯ Sometimes		◯ Often
10. Does your child show objects to you without giving you the object?		◯ Not Yet	◯ Sometimes		◯ Often
11. Does your child wave to greet people?		◯ Not Yet	◯ Sometimes		◯ Often
12. Does your child point to objects?		◯ Not Yet	◯ Sometimes		◯ Often
13. Does your child nod his/her head to indicate *yes*?		◯ Not Yet	◯ Sometimes		◯ Often
**Sounds**					
14. Does your child use sounds or words to get attention or help?		◯ Not Yet	◯ Sometimes		◯ Often
15. Does your child string sounds together, such as : *mama, gaga, papa, dada*?		◯ Not Yet	◯ Sometimes		◯ Often
16. About how many of the following consonant sounds does your child use : *ma, na, ba, da, ga, ka, la, ja, ta, pa?*	◯ None	◯ 1-2	◯ 3-4	◯ 5-8	◯ over 8
**Words**					
17. About how many different words does your child use meaningfully that you recognize (eg.. *am am* na jedzenie, *tote* na kotek etc.)	◯ 0	◯ 1-3	◯ 4-10	◯ 11-30	◯ over 30
18. Does your child put two words together (e.g.: *daj piciu, tata pa pa*)?		◯ Not Yet	◯ Sometimes		◯ Often
**Understanding**					
19. When you call your child's name, does he/she respond by looking or turning toward you?		◯ Not Yet	◯ Sometimes		◯ Often
20. About how many different words or phrases does your child understand without gestures? For example, if you say "where's your tummy," "where's Daddy," "give me the ball," or "come here," without showing or pointing, your child will respond appropriately.	◯ None	◯ 1-3	◯ 4-10	◯ 11-30	◯ over 30
**Object Use**					
21. Does your child show interest in playing with a variety of objects?		◯ Not Yet	◯ Sometimes		◯ Often
22. About how many of the following objects does your child use appropriately: cup, bottle, bowl, spoon, comb or brush, toothbrush, washcloth, ball, toy vehicle, toy telephone?	◯ None	◯ 1-2	◯ 3-4	◯ 5-8	◯ over 8
23. About how many blocks (or rings) does your child stack?	◯ None	◯ 2 blocks	◯ 3-4 blocks		◯ 5 or more
24. Does your child pretend to play with toys (for example, feed a stuffed animal, put a doll to sleep, put an animal figure in a vehicle)?		◯ Not Yet	◯ Sometimes		◯ Often

Do you have any concerns about your child's development? ◯ yes ◯ no If so, please describe them on the back.

Original version: Amy M. Wetherby, Barry M. Prinzant © 2002, Paul H. Brookes Publishing Co, Inc.
Polish version (with the consent of the authors of the original version): Mateusz Sobieski, www.spojrzecwoczy.pl
