# Supplementary material for: Screening accuracy and cut-offs of the Polish version of Communication and Symbolic Behavior Scales-Developmental Profile Infant-Toddler Checklist
Source: PLoS One. 2024 Aug 9;19(8):e0299618. doi: 10.1371/journal.pone.0299618 (PMC11315298; doi:10.1371/journal.pone.0299618)
Supplement: S3 File — Full text of Ethics Committee Approval for conducting this study obtained by the Wroclaw Medical University Ethics Committee. (PDF) [file pone.0299618.s003.pdf]

KOMISJA BIOETYCZNA
przy
Uniwersytecie Medycznym
we Wrocławiu
ul. Pasteura 1; 50-367 WROCŁAW

OPINIA KOMISJI BIOETYCZNEJ Nr KB – 641/2020

Komisja Bioetyczna przy Uniwersytecie Medycznym we Wrocławiu, powołana zarządzeniem Rektora Uniwersytetu Medycznego we Wrocławiu nr 133/XV R/2017 z dnia 21 grudnia 2017 r. oraz działająca w trybie przewidzianym rozporządzeniem Ministra Zdrowia i Opieki Społecznej z dnia 11 maja 1999 r. (Dz.U. nr 47, poz. 480) na podstawie ustawy o zawodzie lekarza z dnia 5 grudnia 1996 r. (Dz.U. nr 28 z 1997 r. poz. 152 z późniejszymi zmianami) w składzie:

prof. dr hab. Jacek Daroszewski (choroby wewnętrzne, endokrynologia, diabetologia)
prof. dr hab. Krzysztof Grabowski (chirurgia)
dr Henryk Kaczkowski (chirurgia szczękowa, chirurgia stomatologiczna)
mgr Irena Knabel-Krzyszowska (farmacja)
prof. dr hab. Jerzy Liebhart (choroby wewnętrzne, alergologia)
ks. dr hab. Piotr Mrzygłód, prof. nadzw. (duchowny)
mgr Luiza Műller (prawo)
dr hab. Sławomir Sidorowicz (psychiatria)
prof. dr hab. Leszek Szenborn, (pediatria, choroby zakaźne)
Danuta Tarkowska (pielęgniarstwo)
prof. dr hab. Anna Wiela-Hojeńska (farmakologia kliniczna)
dr hab. Andrzej Wojnar, prof. nadzw. (histopatologia, dermatologia) przedstawiciel Dolnośląskiej Izby Lekarskiej)
dr hab. Jacek Zieliński (filozofia)

pod przewodnictwem
prof. dr hab. Jana Kornafela (ginekologia i położnictwo, onkologia)

Przestrzegając w działalności zasad Good Clinical Practice oraz zasad Deklaracji Helsińskiej, po zapoznaniu się z projektem badawczym pt.

„Ocena efektywności narzędzi wczesnego wykrywania zaburzeń ze spektrum autyzmu wśród dieci”

zgłoszonym przez **lek. Mateusza Sobieskiego** uczestnika szkoły doktorskiej w Katedrze i Zakładzie Medycyny Rodzinnej Uniwersytetu Medycznego we Wrocławiu oraz złożonymi wraz z wnioskiem dokumentami, w tajnym głosowaniu postanowiła wyrazić zgodę na przeprowadzenie badania w Centrum Medycznym AD-MED. we Wrocławiu; Gabinecie Psychologicznym Natalia Popławska we Wrocławiu; Modelowej Praktyce Lekarzy Rodzinnych we Wrocławiu pod nadzorem dr hab. Marii Magdaleny Bujnowskiej-Fedak **pod warunkiem zachowania anonimowości uzyskanych danych**.

Uwaga: Badanie to zostało objęte ubezpieczeniem odpowiedzialności cywilnej Uniwersytetu Medycznego we Wrocławiu z tytułu prowadzonej działalności:

Pouczenie: W ciągu 14 dni od otrzymania decyzji wnioskodawcy przysługuje prawo odwołania do Komisji Odwoławczej za pośrednictwem Komisji Bioetycznej UM we Wrocławiu

Opinia powyższa dotyczy: projektu badawczego będącego podstawą rozprawy doktorskiej

Wrocław, dnia 21 października 2020 r.

Uniwersytet Medyczny we Wrocławiu
KOMISJA BIOETYCZNA
przewodniczący

prof. dr hab. Jan Kornafel

BW
