# Supplementary material for: Screening accuracy and cut-offs of the Polish version of Communication and Symbolic Behavior Scales-Developmental Profile Infant-Toddler Checklist
Source: PLoS One. 2024 Aug 9;19(8):e0299618. doi: 10.1371/journal.pone.0299618 (PMC11315298; doi:10.1371/journal.pone.0299618)
Supplement: S4 File — Translated from Polish full text of Ethics Committee Approval for conducting this study obtained by the Wroclaw Medical University Ethics Committee. (DOCX) [file pone.0299618.s004.docx]

Bioethical commission

at the Wroclaw Medical University

Pasteura 1 Str. 50-367 Wroclaw

**Opinion of the bioethics commission no. KB - 641/2020**

Bioethics committee at the Wroclaw Medical University established by order of the Rector of the Wroclaw Medical University No. 133/XV R/1017 of December 21, 2017, operating in the manner provided for by the regulation of the Minister of Health and Social Welfare of May 11, 1999 (Journal of Laws No. 47, item 480) on pursuant to the Act on the medical profession of December 5, 1996 (Journal of Laws No. 28 of 1997, item 152, as amended) composed of:

Prof. Jacek Daroszewski (internal diseases, endocrinology, diabetology)

Prof. Krzysztof Grabowski (surgery)

Henryk Kaczkowski PhD (oral and dental surgery)

Irena Knabel-Krzyszowska MSc (pharmacy)

Prof. Jerzy Liebhart (internal diseases, allergology)

Priest Prof. Piotr Mrzygłód (cleric)

Luiza Muller MSc (law)

Sławomir Sidorowicz PhD (psychiatry)

Prof. Leszek Szenborn (pediatrics, infectious diseases)

Danuta Tarkowska (nursing)

Prof. Anna Wiela-Hojeńska (clinical pharmacology)

Andrzej Wojnar PhD (histopathology, dermatology)

Jacek Zieliński PhD (philosophy)

under the leadership of prof. Jan Kornafel (gynecology and obstetrics, oncology)

in compliance with the principles of Good Clinical Practice and the principles of the Declaration of Helsinki after familiarizing themselves with the research project entitled

"Evaluation of the effectiveness of early detection tools for autism spectrum disorders among children"

reported by the Mateusz Sobieski MD, a participant of the doctoral school in Department of Family Medicine, Wroclaw Medical University, and with the documents submitted with the application, decided in a secret vote to consent to the study being carried out online and stationary at the AD-MED Medical Center in Wrocław, the Natalia Popławska Psychological Office in Wrocław; Model Family Physician Practice in Wrocław under the supervision of Professor Maria Magdalena Bujnowska-Fedak, provided that the obtained data is kept anonymous.

Note: This study was covered by the Wroclaw Medical University's civil liability insurance.

Instruction: Within 14 days of receiving the decision, the applicant has the right to appeal to the Appeals Committee through the Wroclaw Medical University's Bioethics Committee.

The above opinion concerns: the research project constituting the basis of the doctoral dissertation.
