# Supplementary material for: Screening accuracy and cut-offs of the Polish version of Communication and Symbolic Behavior Scales-Developmental Profile Infant-Toddler Checklist
Source: PLoS One. 2024 Aug 9;19(8):e0299618. doi: 10.1371/journal.pone.0299618 (PMC11315298; doi:10.1371/journal.pone.0299618)
Supplement: S5 File — (DOCX) [file pone.0299618.s005.docx]

**Sensitivity, specificity of the Polish version of CSBS-DP ITC using tangential method**

Results using the tangential method with different probabilities of ASD occurrence (random, 1:160, and estimated from the sample) are provided in Table 1. For the group aged 9–12 months, the cost of misclassification of a child with ASD as healthy (false negative) was set as equal to 2, in the group of 13–18 months – as equal to 3, in the group of 19–24 months – as 5.

**Table 1**

*Values of sensitivity, specificity, accuracy, positive and negative values for estimated cut-offs using analysis with minimizing expected costs using different presumed prevalences of ASD in Polish population.*

| Presumed prevalence | Estimated  cut-off | Sensitivity | Specificity | Accuracy | PPV | NPV |
| --- | --- | --- | --- | --- | --- | --- |
| *Group II (children aged 9-12 months; N = 131)*  *Assumed misclassification cost = 2* | | | | | | |
| 0.5 (random) | 21 | 0.750 | 0.862 | 0.855 | 0.261 | 0.981 |
| 1:160 | 12 | 0.125 | 1.000 | 0.947 | 1.000 | 0.946 |
| estimated from the sample | 14 | 0.250 | 0.984 | 0.939 | 0.500 | 0.953 |
| *Group II (children aged 13-18 months; N = 242)*  *Assumed misclassification cost = 3* | | | | | | |
| 0.5 (random) | 44 | 1.000 | 0.376 | 0.417 | 0.102 | 1.000 |
| 1:160 | 18 | 0.188 | 1.000 | 0.946 | 1.000 | 0.946 |
| estimated from the sample | 30 | 0.563 | 0.951 | 0.926 | 0.450 | 0.968 |
| *Group IV (children aged 19-24 months; N = 156)*  *Assumed misclassification cost = 5* | | | | | | |
| 0.5 (random) | 54 | 1.000 | 0.109 | 0.160 | 0.064 | 1.000 |
| 1:160 | 30 | 0.111 | 0.993 | 0.942 | 0.500 | 0.948 |
| estimated from the sample | 39 | 0.667 | 0.939 | 0.923 | 0.400 | 0.979 |

It should be noted that the highest values of sensitivity and specificity were achieved using the Youden method, which is consistent with the assumptions of this method. This method assumes the probability of ASD occurrence as 50%, although the probability of ASD could not be unequivocally estimated in the population of children represented by the study sample in the current study. In a situation where the probabilities reported by the WHO were adopted for the analyses, the tangent method gives non-intuitive results and is far too volatile in classifying children into particular risk groups. Usage of cut-off points determined using this method would lead to a situation where the majority of examined children would be referred for further diagnostics. This, in turn, would lead to an increase in unnecessary costs for diagnosis, an increase in the psychological burden for families caused by a potentially unfavorable diagnosis in a child, and a reduction in the availability of specialists in the field of early diagnosis and intervention (because they would have to take care of a larger group of children who would not require this type of assistance). A measurable indicator of the lack of indications for the use of cut-offs determined by the tangent method with minimizing expected costs is a significant decrease in the sensitivity of the questionnaire visible in the ROC analyses.
